# Diabetes

**Published:** 1904-12-10

**Authors:** 


### DIABETES.

**Experimental Glycosuria.**—Glycosuria has been experimentally produced in rabbits by Fischer,[1] by injections of sodium chloride, bromide, or iodide. It was preceded and accompanied by polyuria, which began shortly after the injection and lasted as long as it was continued. The glycosuria is later in appearing and only lasts six to eight hours. The two symptoms vary independently. The glycosuria can be checked by injecting calcium chloride, and increased by sodium citrate, but both substances require half an hour or an hour before their action becomes apparent.

**Diabetic Coma** has been investigated by Douglas,[2] who points out that of the three substances found in the urine—acetone, diacetic acid, and $\beta$-oxybutyric acid—only the last can be considered as causally related to the coma. The two first usually occur together, and in 43 examinations in severe cases acetone was present in 37, and diacetic acid in 32. The latter, moreover, was never present alone. $\beta$-oxybutyric acid was found 19 times in 36 examinations, the cases, mostly severe ones, being 15 in number. In one case the amount of ammonia present was also estimated, and found to correspond with the amount necessary for combining with the $\beta$-oxybutyric acid. Douglas considers the coma to be an acidosis or acid intoxication. The acid for elimination requires a base, and, not obtaining enough soda or potash from the tissues, seizes on the ammonia produced from proteid metabolism, which would normally have become urea. The result of this is to reduce the normal alkalinity of the blood, so that the carbonic acid cannot be combined with and excreted in the lungs. Experimentally the injection of dilute mineral acids has produced symptoms of coma in animals, and large doses of alkalies intravenously and by the mouth have relieved symptoms of coma in man. Dreschfeld and Moore[3] explain acidosis in a similar manner, pointing out that the condition is not due to any toxic property in the acids, but merely to their power of combining with bases and reducing the alkalinity of the blood. They regard the production of ammonia from proteid metabolism as a protective reaction, to save the tissue-fluid alkalies, and note that it occurs much more markedly in carnivorous animals. The source of the acids is doubtful, and may be due to disassimilation of the fatty oxide or the non-nitrogenous part of the proteid molecule; or it may be synthesised from the later products of fat katabolism. It is possible that its presence is due to a failure on the part of the organism to destroy it, owing to the absence of some product of carbohydrate metabolism—perhaps glycuronic acid. The authors divide cases of acidosis into three classes: (1) Those occurring in diabetes; (2) those secondary to starvation or inanition; (3) those in which no causative factor can be found. They quote three cases: one of the last type; one associated with "cyclic vomiting," in which there was but slight inanition; and one associated with uncompensated mitral disease. All occurred in women. Rolleston and Tebbs[4] also point out the frequent occurrence of diacetic acid in the urine of women suffering from starvation. In 44 cases of gastric ulcer, 40 occurred in women, and of these all but five showed diacetic acid in the urine, and the reaction appeared early and was persistent; in the four men it did not occur at all, or only after long periods of starvation. The acute or chronic character of the ulcer did not appear to affect the reaction. In other gastric conditions the reaction was almost invariably absent while food was being given by the mouth. In three cases of alcoholic gastritis (one of which was a male) it appeared early, and seemed due to some metabolic disturbance produced by alcohol. In other conditions in which the intestinal tract was mainly involved, the reaction for diacetic acid was much more frequently observed in females, while apart from these disorders it was very rare. The only disease in which males gave a reaction as often as females was pneumonia. The other theories as to the production of coma in diabetes are given by Douglas.[5] Thus Sternberg has suggested $\beta$-amidobutyric acid, which is known to cause coma and laboured breathing, and by hydration yields $\beta$-oxybutyric acid and ammonia. Methylamine and paraldehyde have also been suggested, and also a toxin derived from the destruction of albuminous matter.

**Pancreatic Lesions.**—In dealing with pancreatic diabetes Pearce[6] points out that the structure and development of the islands of Langerhans seem to show that they have an independent function. They arise from the primary acini, and when developed possess an abundant vascular supply, which seems to indicate their connection with the composition of the blood. Edsall[7] considers that carbohydrate metabolism is connected with other tissues besides the pancreas, and that the latter probably acts indirectly by influencing the production of a glycolytic ferment elsewhere. Hale White[8] notes that out of 6,708 autopsies at Guy's Hospital 2 per cent. showed naked-eye changes in the pancreas; 19 of these were cases of small atrophic pancreas, and, of these 19, 16 had suffered from diabetes. Moreover, a quarter of all patients dying of diabetes were found to have a small atrophic pancreas *post mortem*. Oser and Hausmann gave similar results, both regarding atrophy as the commonest lesion of the pancreas in diabetes. Too much importance, however, should not be attached to the hyperglycæmia associated with pancreatic lesions in diabetes It is only one link in a chain which included very many disordered metabolic processes, and, moreover, is not the one which killed the patient. Hale White considers that under the heading of diabetes a number of different conditions are included.

[1] Med. Rec., March 26, 1904, p. 501. [2] Brit. Med. Jour., Dec. 26, 1903, p. 1629. [3] Med. Chron., May, 1904, p. 71. [4] Brit. Med. Jour., July 16, 1904, p. 114. [5] *Loc. cit.* [6] Med. Rec., May 21, 1904, p. 817. [7] Ibid. [8] Lancet, March 26, 1904, p. 870.

(*To be continued.*)

